# From the Editor’s Desk

**Published:** 2015

**Authors:** PJ Commerford

**Affiliations:** Editor-in-Chief

Healthcare professionals often label patients as ‘non-compliant’ when prescribed therapies seem to fail to be effective. This is often used as a derogatory term of criticism of patient behaviour, as though patients are personally liable for their lack of response and continued ill health. Those of us who have experienced having been prescribed long-term medication, requiring multiple daily doses, will readily admit to the difficulty of complying fully with complex regimens despite the best will in the world. The article by Osamor in this issue (page 29) explores the importance of social support in the management of a chronic disease in Africa and confirms the importance of such support in ensuring compliance with prescribed medication. Given the evidence from elsewhere that familial and social support is of importance in lessening the likelihood of developing cardiovascular disease and increasing the likelihood of compliance with treatment, the results are not surprising, and are important for those teaching medicine and planning treatment programmes in Africa. Hopefully the results will stimulate further African research in this important area.

Coronary artery bypass grafting (CABG) has produced excellent symptomatic relief from angina for many patients and enhanced survival in selected sub-groups. Symptomatic relief is inevitably time-limited by durability of the venous conduits. When symptoms recur due to vein graft failure and a percutaneous intervention is not feasible, patients and clinicians face a difficult dilemma, particularly if there is a patent left internal thoracic artery anastomosis to the left anterior descending coronary artery that may be jeopardised at the time of repeat median sternotomy. Such patients are inevitably older with multiple co-morbidities. Duvan and colleagues (page 25) report their experience with redo off-pump CABG via a posterolateral thoracotomy to access branches of the circumflex coronary artery. This report serves as a timely reminder of an alternative revascularisation strategy that may well be acceptable to both patients and referring cardiologists when severe symptoms persist despite optimal medical therapy.

The precise relationship between obesity and coronary disease remains unclear. Zand-Parsa and colleagues (page 13) add a new level of complexity by demonstrating that obesity, as determined by waist-to-hip ratio (WHR), was correlated with severity of coronary artery disease by two independent scoring systems, whereas body mass index (BMI) was not. Clinicians will be aware of considerable ethnic variation in patterns of distribution of adipose tissue and this is not always considered in comparisons of BMI and WHR. It may, in part, account for some discrepancies, and the establishment of regional norms may be necessary.

Laboratory experimental work by Burma (page 4), using pre-constricted internal mammary artery (IMA) rings in a tissue bath, showed that leptin caused relaxation of these arterial segments. These findings led the authors to raise the intriguing hypothesis that obese subjects who had a left IMA bypass graft would actually have better (anterior wall) myocardial perfusion compared to non-obese subjects. The risks and benefits of obesity in patients with coronary disease are far from settled!

